# The lipid profile of HIV-infected patients receiving antiretroviral therapy in a rural Cameroonian population

**DOI:** 10.1186/1471-2458-14-236

**Published:** 2014-03-07

**Authors:** Cavin Epie Bekolo, Modestine Becker Nguena, Leonard Ewane, Patrick Sylvestre Bekoule, Basile Kollo

**Affiliations:** 1 Centre Médical d’Arrondissement de Baré, P.O. Box 628 Nkongsamba, Cameroon; 2 Centre d’Enseignement Spécialisé des Techniques d’Analyses Médicales de Yaounde, P.O. Box 20179 Yaounde, Cameroon; 3 The Central African Field Epidemiology and Laboratory Training Program, University of Yaounde 1, Yaounde, Cameroon; 4 Regional Hospital of Nkongsamba, P.O. Box 03, Nkongsamba, Cameroon; 5 Department of Public Health, University of Douala, P.O. Box 2701, Douala, Cameroon

**Keywords:** HIV, Antiretroviral therapy, Blood lipids, Cameroon

## Abstract

**Background:**

Long term use of antiretroviral therapy (ART) in persons living with human immunodeficiency virus (PLWHIV) is associated with disturbances in blood lipids which should be monitored. More data on such disturbances are needed in Cameroon to persuade the country program to institute their routine monitoring. We then sought to determine the prevalence and timing of dyslipidaemia in PLWHIV and receiving ART in a predominantly rural Cameroonian setting.

**Methods:**

A cross-sectional study conducted between August and October 2012 in HIV-infected persons aged 15 years or more and receiving first-line ART for at least six months at The Nkongsamba Regional Hospital in Cameroon. Lipid assays were carried out by enzymatic-linked colorimetric methods. A multiple logistic regression model was used to assess for factors related to dyslipidaemia.

**Results:**

Included were 114 participants of whom 83 (72.8%) were females. Their median age was 43 years (IQR: 36–51) and their median CD4 count was 436 cells/μl (IQR: 275–585) after a median duration on ART of 36 months (IQR: 12–60). The prevalence of dyslipidaemia was 70.2%. Hypercholesterolaemia was observed in 34 (29.8%) patients. One-third of them had a high LDL-cholesterol level (LDL-c ≥ 130 mg/dl). Hypertriglyceridaemia (TG ≥ 150 mg/dl) was present in 59 (51.8%) cases. The proportion of patients with a low HDL-cholesterol (HDL-c < 40 mg/dl) was 18.4% while those with a ratio of TC/HDL-c ≥ 5 were about 16.7%. A duration of 2–4 years on ART (adjusted Odd Ratio, aOR = 5.22, 95% CI: 1.43-19.06, p = 0.01), current smokers (aOR = 15.94, 95% CI: 1.13-225.61, p = 0.04) and a concurrent metabolic disease (aOR = 12.54, 95% CI: 1.02-153.86, p = 0.48) were independently associated with pro-atherogenic LDL-c values. Alcohol users had a more friendly LDL-c profile (aOR = 0.24, 95% CI: 0.07-0.74, p = 0.01).

**Conclusion:**

The study has demonstrated a high prevalence of dyslipidaemia in HIV-patients receiving first-line ART in a predominantly rural setting of Cameroon. There is a need for the country HIV program to institute laboratory monitoring of blood lipids in patients over two years on first line ART with a focus on smokers.

## Background

At the end of 2012, an estimated 35.3 million people were living with human immunodeficiency virus (HIV) worldwide [1]. Sub-Saharan Africa, a region with only 12% of the global population remains the region most heavily affected by HIV accounting for about 70% of the global burden of HIV. The scaling up of antiretroviral therapy (ART) in low- and middle-income countries has transformed national AIDS (acquired immune deficiency syndrome) responses and generated broad-based health gains. Globally, from 1996 to 2012, a total of 6.6 million AIDS-related deaths have been averted, including 5.5 million deaths in low- and middle-income countries due to increased access to antiretroviral therapy (ART) [1]. However, long-term use of ART is related to metabolic (dyslipidaemia, insulin resistance, and diabetes) and cardiovascular complications [2,3]. Antiretroviral therapy can induce raised levels of total cholesterol (TC), low density lipoprotein-cholesterol (LDL-c) and triglycerides (TG), and variable effects on high density lipoprotein-cholesterol (HDL-c) levels [4]. In a large cross-sectional study, the prevalence of hypercholesterolemia (>6.2 mmol/L), hypertriglyceridemia (>2.3 mmol/L), and low HDL-cholesterol (<0.9 mmol/L) was 10 to 27 per cent, 23 to 40 per cent, and 19 to 27 per cent, respectively, depending on the antiretroviral regimen [5]. The treatment options available for the management of dyslipidaemia in HIV-infection are similar to those in the general population with an additional option of switching to a different antiretroviral class with a friendlier lipid profile [3].

Cameroon with an estimated population of 19.4 million in 2010 [6] had an HIV prevalence rate in 2011 of 4.3% down from 5.5% in 2004 [7]. The ART coverage increased from 36.5% in 2010 to 49.6% in 2011 [8]. HIV infection in ART-naïve Cameroonians has been shown to be associated with dyslipidaemia characterised by a significant decrease in total oxidant ability (TAA), LDL-c, HDL-c and TC; and increased malondialdehyde (MAA) and lipid peroxidation indices (LPI) than in their seronegative counterparts [9-12]. Routine monitoring of serum lipids in patients on ART is not a common practice in the country. In 2011, however, following recommendations from WHO 2010 guidelines, stavudine (D4T) was phased out due to its metabolic toxicity amongst others [13]. One study in the country on ART-associated dyslipidaemia, had found that ART compounded the effects of HIV by increasing lipid oxidation [9] while another demonstrated that patients on first-line ART had high levels of total cholesterol, LDL-c and high TC/HDL-c ratio compared with ART-naive patients [14]. These studies were carried out in the urban setting and recommended that lipid profile and other cardiovascular risk factors should be monitored in patients on ART so that any untoward effects of treatments can be optimally managed. Their recommendations are yet to be applied in full.

A small study carried out in the Littoral Region of Cameroon, reported higher levels of TC, TG and LDL-c with lower levels of HDL-c in the urban than in the rural area. These rural–urban differences in lipid profile were attributed to differences in dietary habits. The traditional diets of rural populations are rich in many vegetables and season fruits that enhance hepatic cholesterol clearance allowing for a less atherogenic lipid profile in the rural population [15].

However, data on dyslipidaemia among HIV-infected patients using ART in a rural setting are virtually non-existent. We carried out an opportunistic cross sectional study to determine the prevalence of dyslipidaemia in rural Cameroon using in service data from an HIV clinic wherein all patients have an unknown lipid profile. We hoped that the findings of this study would inform health workers at the point of care and program managers on reviewing the guidelines for laboratory monitoring of ART-associated toxicity.

## Methods

### Study site

The study was conducted at the Regional Hospital of Nkongsamba in the Moungo Division of the Littoral Region of Cameroon. It is a second level of reference public health facility with a catchment area of over 321,295 inhabitants [6] most of whom are rural peasant farmers. Users of the clinic come from the city of Nkongsamba obviously but massively constitute of referrals from the rural municipalities of Melong, Bare-Bakem, Nlonako, Manjo, Loum, Njombe-Penja, Mbanga, Bonalea and Dibombari that make up the Moungo Division; equally from neighbouring rural areas of Bangem and Tombel in the South-West Region and from the rural municipalities of Kekem and Santchou in the West Region of Cameroon. The clinic was established in 2005 and offers voluntary HIV counselling and testing (VCT), ART and limited community outreach services to patients on ART.

### Ethical aspects

Ethical approval was obtained from the Centre for Medical Laboratory Technicians in Yaounde, Cameroon (Centre d’Enseignement Spécialisé des Techniques d’Analyses Médicales de Yaounde). Site permission to conduct the study was provided by The Director of The Nkongsamba Regional Hospital. A written informed consent from each of the participants or legal representatives was duly obtained.

### Participants and study design

We carried out a cross sectional study involving HIV-infected patients of age 15 years and above receiving ART and returning for their six-monthly laboratory check-up between August 2012 and October 2012. The study was opportunistic with a sampling method of convenience in the sense that, we took advantage of patients turning up voluntarily for their routine and subsidised semester laboratory monitoring tests to which we added the lipid measuring tests according to our constraint budget. Lipid profile had never been included in the subsidised laboratory kit nor had been part of a routine measurement in this centre. Prior to sample collection, sociodemographic, clinical, laboratory and treatment related variables of interest were collected by interviews and from medical records using a structured questionnaire. Blood samples were collected in the mornings after an overnight fasting and centrifuged at 3000 g per minute for 30 minutes. The serum obtained was stored at 4°C and later used for lipid assays using a spectrophotometer (Helios Gamma, UVG 123019, Thermo Electron Corporation, England) according to the kit material referenced [Chronolab (Systems; S.L) and QCA (Quimica Clinica Aplicada; S.A].

TC concentration was determined using colorimetric enzymatic techniques based on the successive action of cholesterol oxidase and peroxidase; HDL-c concentration in the serum supernatant was determined by the same process after the precipitation of very low density lipoprotein (VLDL) cholesterol, LDL-c and chylomicrons. Results were calculated using the formula: TC (g/l) or HDL-c (mg/dl) concentrations = (Optical Density _at 500nm_ of sample ÷ Optical Density _at 500nm_ of standard) × Concentration of standard (essentially as recommended by the manufacturer in the kits with all units later converted to mg/dl were necessary). LDL-c concentration was determined using the formula of Friedewald et al. [16]: LDL-c (mg/dl) = TC (mg/dl)-[HDL-c (mg/dl)-Triglycerides (mg/dl)/5].

### Data analysis

Data analyses were performed using Stata® 12.1(StataCorp LP, TX77845, USA). The data set was checked for logical inconsistencies, illegal codes, omissions and improbabilities by tabulating, summarising, describing and plotting variables. Missing observations were excluded where they constituted a small random proportion but were included if they were found to be differential amongst subgroups.

Our outcomes of interest included the lipid parameters: TC, HDL-c, LDL-c, TG and TC/HDL-c ratio. In accordance with the United States National Cholesterol Education Program, Adult Treatment Panel III (NCEP-ATP III) guidelines, abnormal lipid profile was defined as TC ≥ 200 mg/dl, HDL-c < 40 mg/dl, LDL-c ≥ 130 mg/dl, TG ≥ 150 mg/dl and TC/HDL-c ratio ≥ 5 [17]. Putative risk factors for dyslipidaemia included: age, sex, CD4 count, ART regimen, duration of and adherence to ART, smoking, alcohol use, presence of metabolic and/or cardiovascular disease. No factor was considered as an *a priori* confounder or effect modifier.

Summary statistics were presented as proportions for categorical variables and as means (standard deviations) or medians (IQR: Inter-quartile Range) for continuous variables. Pearson Chi-square analyses were used to examine the difference in proportion of abnormal lipid parameters between the various categories of an explanatory variable. A multivariable logistic regression model was set up to screen for factors independently associated with a pro-atherogenic lipid profile. Adjusted odd ratios (aOR) and their 95% confidence intervals (95%CI) obtained. The P-values for hypotheses testing were calculated from Wald or likelihood ratio tests (LRT).

## Results

### Participants

The study included patients receiving ART and returning to the clinic between August 2012 and October 2012 for their routine biannual laboratory monitoring. A total of 200 patients were recruited of which 86 were children below 15 years old who were excluded from the study.

The results of the 114 adults are reported. Of these, 83 (72.8%) were females (Table 1). Their median age was 43 years (IQR: 36–51) and their median CD4 count was 436 cells/μl (IQR: 275–585) after a median duration on ART of 36 months (IQR: 12–60). With lamivudine (3TC) constantly present in all the first line triple-combination antiretroviral regimens, zidovudine (AZT) completed the nucleotide reverse transcriptase inhibitor (NRTI) backbone in 75 (65.8%) cases while nevirapine (NVP) in 60 (52.6%) patients was the preferred non-nucleotide reverse transcriptase inhibitor (NNRTI) option. Up to 49 (43.6%) patients reported using alcohol, but only six could declare that they were current smokers. Metabolic and cardiovascular diseases in 16 subjects were rare conditions amongst the participants (< 10%). Ten of them (9%) reported being on a diet while only five (4.5%) had declared ever interrupted their antiretroviral treatment.

**Table 1:** Baseline characteristics of the study population

**Variables**	**Number (%)**
**Gender**	
Male	31 (27.2)
Female	83 (72.8)
Total	114 (100)
**Age group (years)**	
15 – 24	2 (1.7)
25 – 34	23 (20.2)
35+	89 (78.1)
Total	114 (100)
**CD4 count**	
<350	42 (36.8)
350 – 499	32 (28.1)
500+	40 (35.1)
Total	114 (100)
Duration of ART*(years)	
<2	38 (34.2)
2 – 4	30 (27.0)
5+	43 (38.7)
Total	111 (100)
NRTI** regimen	
AZT-based	75 (65.8)
D4T-based	4 (3.5)
TDF-based	35 (30.7)
Total	114 (100)
NNRTI** regimen	
EFV-based	54 (47.4)
NVP-based	60 (52.6)
Total	114 (100)
**Smoking**	
No	106 (94.6)
Yes	6 (5.4)
Total	112 (100)
**Alcohol intake**	
No	63 (56.2)
Yes	49 (43.5)
Total	112 (100)
**Heart condition**	
No	104 (93.7)
Yes	7 (6.3)
Total	111 (100)
**Metabolic condition**	
No	102 (91.9)
Yes	9 (8.1)
Total	111 (100)
**Special diet**	
No	101 (91.0)
Yes	10 (9.0)
Total	111 (100)
**ART interruption**	
No	107 (95.5)
Yes	5 (4.5)
Total	112 (100)

* ART = antiretroviral therapy, **NRTI = nucleotide reverse transcriptase inhibitors, *** NNRTI = non-nucleotide reverse transcriptase inhibitor, AZT = zidovudine, 3TC = lamivudine, TDF = tenofovir, D4T = stavudine, EFV = efavirenz, NVP = nevirapine.

### Lipid profile of participants

**Figure 1:**
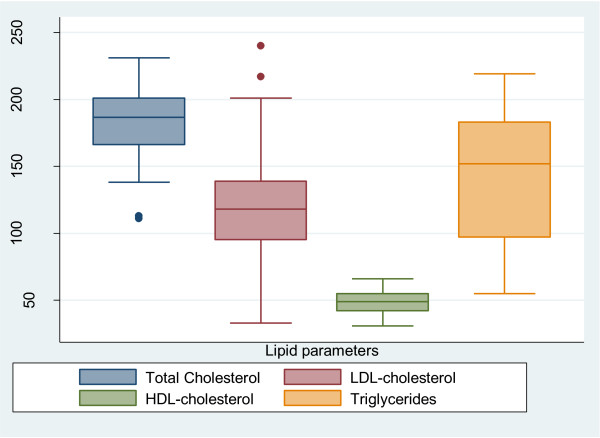
Lipid profile of the study population.

**Figure 2:**
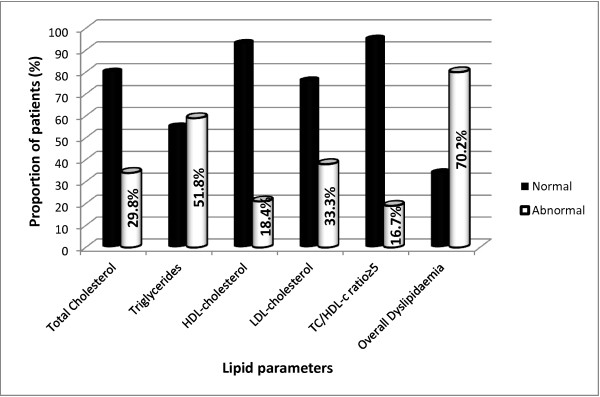
Prevalence of dyslipidaemia in HIV-infected patients receiving HAART.

Figures 1 shows that, the median serum concentration of total cholesterol was 187 mg/dl (IQR: 166–201) with a prevalence of hypercholesterolaemia of 29.8% (Figure 2). One-third of the patients had a high LDL-c and the median serum LDL-c concentration was 118 mg/dl (IQR; 95–139). Hypertryglyceridaemia was present in 59 (51.8%) cases and the median triglyceride level was 152 mg/dl (IQR: 97–183). The proportion of patients with a low HDL-c was 18.4% and that with a high ratio of TC/HDL-c was 16.7%. The number of patients with an abnormal lipid level was 80 (70.2%).

### Factors associated with poor lipid profiles

In multivariable logistic regression analysis (Table 2), there was some evidence that being on ART for two to four years was associated with a higher LDL-c level (aOR = 5.22, 95% CI: 1.43-19.06, p = 0.01) but above four years on ART, the evidence was very weak (aOR = 1.59, 95% CI: 0.45-5.57, p = 0.4). While stavudine and tenofovir (TDF) appeared to be associated with a poorer lipid profile than zidovudine, efavirenz was likely to have a friendlier lipid profile than nevirapine. Smoking was linked to very high levels of LDL-c (aOR = 15.94 95% CI: 1.13-225.61, p = 0.04) as well as very high levels of TC and TG. Meanwhile alcohol consumption was associated with low LDL-c level (aOR = 0.24, 95% CI: 0.07-0.74, p = 0.01) as well as lower levels of TC, TG and higher levels of HDL-c. Patients with existing metabolic conditions were associated with very high levels of LDL-c (aOR = 12.54, 95% CI: 1.02-153.86, p = 0.048) and higher levels of TC, TG but with higher levels of HDL-c. Being male, young or having a CD4 count < 350 cells/μl was generally associated with a better lipid profile though there was little or no evidence to support these relationships.

**Table 2:** Factors associated with dyslipidaemia from a multiple logistic regression model

**Variables**	**TC ≥ 200 mg/dl**	**LDL-c ≥ 130 mg/dl**	**TG ≥ 150 mg/dl**	**HDL-c < 40 mg/dl**	**TC/HDL-c ratio ≥5**
	**Adjusted OR (95%CI)**	**Adjusted OR (95%CI)**	**Adjusted OR (95%CI)**	**Adjusted OR (95%CI)**	**Adjusted OR (95%CI)**
**Sex**					
Male	1	1	1	1	1
Female	2.11 (0.64-6.95)	0.44 (0.14-1.38)	1.47 (0.53-4.08)	2.55 (0.51-12.64)	3.25 (0.56-18.92)
P-value	0.22	0.16	0.45	0.25	0.19
**Age group (years)**					
15 – 24	1	1	1	1	1
25 – 34	0.91 (0.19-4.28)	1.00 (0.03-31.54)	1.20 (0.27-5.30)	1.47 (0.21-10.40)	1.42 (0.19-10.76)
35+	NA	0.28 (0.01-11.70)	NA	NA	NA
P-value	0.56	0.07	0.81	0.70	0.48
**CD4 count**					
<350	1	1	1	1	1
350 – 499	1.12 (0.30-4.14)	1.30 (0.38-4.48)	1.23 (0.38-3.89)	1.24 (0.27-5.66)	1.23 (0.33-8.33)
500+	2.45 (0.73-8.26)	0.77 (0.22-2.64)	0.97 (0.33-2.86)	0.57 (0.11-2.76)	1.28 (0.25-6.59)
P-value	0.56	0.07	0.46	0.65	0.48
**Time on ART(years)**					
<2	1	1	1	1	1
2 – 4	2.16 (0.64-7.33)	**5.22 (1.43-19.06)**	0.51 (0.16-1.59)	1.71 (0.34-8.48)	1.65 (0.33-8.33)
5+	1.11 (0.33-3.75)	1.59 (0.45-5.57)	0.66 (0.22-1.91)	2.49 (0.49-12.51)	1.67 (0.30-9.20)
P-value	0.56	0.07	0.46	0.65	0.48
**NRTI regimen**					
AZT-based	1	1	1	1	1
D4T-based	2.17 (0.22-21.47)	1.47 (0.08-25.46)	5.29 (0.44-64.26)	5.62 (0.33-95.76)	6.79 (0.40-116.57)
TDF-based	1.89 (0.62-5.80)	1.25 (0.38-4.06)	2.28 (0.79-6.63)	0.74 (0.17-3.26)	1.37 (0.31-6.03)
P-value	0.56	0.07	0.46	0.65	0.48
**NNRTI regimen**					
EFV-based	1	1	1	1	1
NVP-based	1.27 (0.42-3.81)	1.20 (0.38-3.76)	0.80 (0.29-2.24)	3.96 (0.85-18.52)	4.09 (0.85-19.73)
P-value	0.67	0.76	0.67	0.08	0.08
**Smoking**					
No	1	**1**	1	NA	NA
Yes	5.84 (0.51-67.18)	**15.94 (1.13-225.61)**	6.06 (0.44-83.99)	NA	NA
P-value	0.16	**0.04**	0.18		
**Alcohol intake**					
No	1	**1**	1	1	1
Yes	0.50 (0.18-1.41)	**0.24 (0.07-0.74)**	0.99 (0.40-2.47)	0.70 (0.21-2.42)	0.58 (0.15-2.17)
P-value	0.19	**0.01**	0.99	0.58	0.42
**Heart condition**					
No	1	NA	1	NA	NA
Yes	0.47 (0.04-6.15)	NA	0.25 (0.02-3.24)	NA	NA
P-value	0.56		0.29		
**Metabolic condition**					
No	1	**1**	1	1	1
Yes	1.42 (0.21-9.79)	**12.54 (1.02-153.86)**	2.97 (0.38-22.94)	0.67 (0.06-8.08)	1.02 (0.07-14.69)
P-value	0.72	**0.048**	0.30	0.75	0.99
**Special diet**					
No	1	1	1	1	1
Yes	3.36 (0.56-20.20)	4.19 (0.42-42.07)	1.81 (0.28-11.69)	3.95 (0.45-34.50)	1.06 (0.08-14.17)
P-value	0.19	0.22	0.53	0.22	0.97
**ART interruption**					
No	1	1	1	NA	NA
Yes	0.75 (0.05-11.45)	0.89 (0.03-26.83)	7.37 (0.36-149.65)	NA	NA
P-value	0.84	0.95	0.19		

* ART = antiretroviral therapy, **NRTI = nucleotide reverse transcriptase inhibitors, ***NNRTI = non-nucleotide reverse transcriptase inhibitor, AZT = zidovudine, 3TC = lamivudine, TDF = tenofovir, D4T = stavudine, EFV = efavirenz, NVP = nevirapine, NA = not applicable.

## Discussion

The study has demonstrated a high prevalence of dyslipidaemia (70.2%) in HIV-patients receiving first-line ART in a predominantly rural population of Cameroon. Hypertriglyceridaemia (51.8%), raised levels of LDL-c (33.3%) and hypercholesterolaemia (29.8%) were the most common forms of dyslipidaemia. The duration on ART, smoking, alcohol use and the presence of a concurrent metabolic disease were significantly associated with high LDL-c values. LDL-cholesterol is a pro-atherogenic marker.

Though high, the prevalence of dyslipidaemia in our setting is lower to the reported rate of 82.3% in patients using ART in an urban population of Southern Ethiopia [18] and to that observed in Dar es Salaam, Tanzania where the prevalence was 76% in ART-naive patients [19] but much more higher than prevalence rates observed in the developed world [5]. The prevalence rates of hypertriglyceridaemia and high LDL-c levels in our study were comparable to those observed in Southern Ethiopian study (55.8% and 33.6% respectively) [18] but were respectively higher (43.5%) and lower (46.4%) in the urban Cameroon setting [14]. The prevalence rates of hypercholesterolaemia and low HDL-c concentration in our study were lower than those reported in these studies. In the general population of Cameroon, dyslipidaemia was observed to be more prevalent in the urban than in the rural setting [15]. It is however difficult to ascertain whether the prevalence and pattern of dyslipidaemia in HIV-infected population in rural area is different from that in an urban settings because dyslipidaemia in HIV-infected individuals is a complex condition, with multiple contributing factors including the HIV virus itself, individual genetic characteristics and antiretroviral therapy-induced metabolic changes [20].

Women were associated with a greater risk of lipid disturbances than men though our study lacked the evidence to support this association. Increasing the power of our study and adjusting for body mass index could have improved our probability of detecting the sex difference. However, this finding is coherent with current knowledge that women experience more ART-induced (metabolic) adverse effects than men [21].

As age increases, pro-atherogenic lipid parameters also increase [19]. In our study, though it appeared that younger persons below the age of 25 years were at lower risk of dyslipidaemia, there was no pattern for the association between age and abnormal lipid profile. Poor categorisation of age groups in our study might have been responsible for the inability to detect any significant relationship.

Severe immune suppression (low CD4 count) has been associated with dyslipidaemia in ART-naive HIV infected persons [19,22,23]. However, in a cohort of persons doing well on highly active antiretroviral therapy (HAART) with a sufficiently improved mean CD4 count, there is little variability in CD4 count of the group and therefore a difference in dyslipidaemia cannot be attributed to differences in CD4 counts. The latter is the case in our study and in other studies involving patients on HAART [14,18].

HAART is associated with a cardio-protective lipid profile in the short term [24] because after initiation of ART, lipid levels return to baseline levels but soon they rise above pre-sero-conversion levels in the long term [25]. In our cohort, patients with duration on ART above two years were significantly associated with a poor lipid profile. It might be therefore reasonable to recommend that monitoring of lipid profile should be instituted after two years on first line ART in Cameroon. Current guidelines of The National AIDS Control Committee of Cameroon do not allow for routine monitoring of lipid parameters for patients receiving first line ART [26]. Though an earlier study in Cameroon by Yone et al. did recommend monitoring of lipid profile in patients on first line ART, the timing for this laboratory assessment was not mentioned.

Stavudine is known to be associated with a significant increase in lipid parameters compared with either zidovudine [27,28] or tenofovir [29]. Our data suggested a similar comparison. Following the 2010 WHO Guidelines [13], most patients on stavudine had already been switched to either zidovudine or tenofovir at the time of the study. The effect of this intra- class switching has not been accounted for but the likely impact on the odd ratios would be a bias towards unity because the switching was independent of this study.

A head-to-head comparison of efavirenz with nevirapine in initial regimens demonstrated a more favourable lipid profile for nevirapine at 48 weeks [30]. Our data showed the contrary and the likely explanation is that, in our cohort, regimens were selectively prescribed to certain groups of patients according to their immune status or the presence of tuberculosis or pregnancy. This selection bias could account for the difference observed.

A comprehensive meta-analysis by Craig et al. has demonstrated that compared with non-smokers, cigarettes smokers had poorer lipid profiles both in the general population [31] and even amongst the HIV-positive population [32]. Though the proportion of current smokers in our sample population was very small and made up exclusively of men, the association is in agreement with current knowledge and in this study smokers even had the highest odds of dyslipidaemia than any other subgroup. Information bias associated with under-reporting of undesirable lifestyles is likely to be the case here but such a big odd ratio is also unlikely to be a chance finding. Smoking cessation would thus be strongly recommended.

The proportion of alcohol users in our study sample was high just like in the general Cameroon population. Our data suggest that alcohol consumers have better lipid profiles than non-drinkers. It is however not known what unit of daily intake was associated with a favourable lipid profile because we could not determine the quantity consumed per participant. With women in a rural African setting making up the majority of our study population, it is plausible that users of alcohol would be mostly low-to-moderate drinkers. Alcohol consumption should however be discouraged because its deleterious effects on ART adherence and HIV progression [33] and on other cardiac-metabolic disorders [34] do outweigh this supposedly positive effect on lipid parameters. Incidentally, patients with existing metabolic disorders (all of whom were sufferers of type 2 diabetes mellitus) have been shown to have very high odds of dyslipidaemia (LDL-c) in this study.

Our study had a couple of draw-backs that may impact on its quality. The sample size was small so much so that the precision of our odd ratios was low and we lacked the power to detect significant differences we hoped to. Its opportunistic nature had somehow introduced selection bias because patients turning up for their routine check-up might be different from those defaulting. The cross-sectional design made it impossible to assume any causality.

## Conclusion

In conclusion, dyslipidaemia is highly prevalent in patients receiving HAART but with no peculiarities in rural Cameroon. Given that longevity on ART has been improved making HIV a chronic manageable disease, there is a need for the country HIV program to institute laboratory monitoring of lipids for patients in their third year on first line ART with a special attention to be focused on smokers.

## Competing interests

The authors declare that they have no competing interests.

## Authors’ contributions

CEB: Project design, data analysis and interpretation, drafting of manuscript, MBN: Project conception, data collection and laboratory measurements, LE: laboratory measurements and quality control, critical review of manuscript, PSB: Additional information and correction of the revised version of manuscript, BK: critical review, proof-reading and approval for publication, all authors read and approved the final manuscript.

## Authors’ information

CEB (MD, MSc, DLSHTM) is Chief Medical Officer of the Bare Sub-divisional Medical Centre and a visiting physician at the HIV Clinic of the Nkongsamba Regional Hospital in Cameroon. MBN is a recent laboratory scientist graduate of the Centre d’Enseignement Spécialisé des Techniques d’Analyses Médicales de Yaounde, Cameroon. LE (B.Sc) is a Senior Medical Laboratory Scientist at The Regional Hospital of Nkongsamba in Cameroon and a current MSc candidate for The Central African Field Epidemiology and Laboratory Training Program at The University of Yaounde 1, Cameroon. PSB (MD) is a surgeon and the Director of the Nkongsamba Regional Hospital. BK (MD, Sc.D.) is a The Head of The Department of Public Health at The University of Douala, Cameroon, Board Chairman of The Nkongsamba Regional Hospital and Head of The Nkongsamba Urban Council.

## Pre-publication history

The pre-publication history for this paper can be accessed here:

http://www.biomedcentral.com/1471-2458/14/236/prepub
